# Myeloid Derived Suppressor Cells Interactions With Natural Killer Cells and Pro-angiogenic Activities: Roles in Tumor Progression

**DOI:** 10.3389/fimmu.2019.00771

**Published:** 2019-04-18

**Authors:** Antonino Bruno, Lorenzo Mortara, Denisa Baci, Douglas M. Noonan, Adriana Albini

**Affiliations:** ^1^Scientific and Technology Pole, IRCCS MultiMedica, Milan, Italy; ^2^Laboratory of Immunology and General Pathology, Department of Biotechnology and Life Sciences, University of Insubria, Varese, Italy; ^3^School of Medicine and Surgery, University of Milano-Bicocca, Monza, Italy

**Keywords:** myeloid derived suppressor cell (MDSC), natural killer cells (NK cells), angiogenesis, cytokines, tumor microenvironment, decidua

## Abstract

Myeloid-derived suppressor cells (MDSCs) contribute to the induction of an immune suppressive/anergic, tumor permissive environment. MDSCs act as immunosuppression orchestrators also by interacting with several components of both innate and adaptive immunity. Natural killer (NK) cells are innate lymphoid cells functioning as primary effector of immunity, against tumors and virus-infected cells. Apart from the previously described anergy and hypo-functionality of NK cells in different tumors, NK cells in cancer patients show pro-angiogenic phenotype and functions, similar to decidual NK cells. We termed the pro-angiogenic NK cells in the tumor microenvironment “tumor infiltrating NK” (TINKs), and peripheral blood NK cells in cancer patients “tumor associated NK” (TANKs). The contribution of MDSCs in regulating NK cell functions in tumor-bearing host, still represent a poorly explored topic, and even less is known on NK cell regulation of MDSCs. Here, we review whether the crosstalk between MDSCs and NK cells can impact on tumor onset, angiogenesis and progression, focusing on key cellular and molecular interactions. We also propose that the similarity of the properties of tumor associated/tumor infiltrating NK and MDSC with those of decidual NK and decidual MDSCs during pregnancy could hint to a possible onco-fetal origin of these pro-angiogenic leukocytes.

## Introduction

The tumor microenvironment (TME) shapes the fate of tumor onset and progression, by regulating cell growth, invasiveness, immune escape, dissemination and clinical outcome (1, 2). It is now clear that the contribution of tissue-resident immune cells in supporting or limiting tumor growth, metastasis and resistance to therapies has a master role (2, 3). The immune cell effector's capabilities of reaching, recognizing, and eliminating the tumor targets is conditioned by other microenvironment cells and determinants, turning the immune system from early strategic line of defense, into a pro-tumor weapon (1–3). TME employs multiple mechanisms to switch off the anti-tumor functions of immune cells: it can destabilize and polarize the innate cell compartment (macrophages, neutrophils and dendritic cells as well as innate lymphoid cells), the adaptive immunity (T and B lymphocytes), stromal cells (cancer-associated fibroblasts) or endothelial cells (tumor associated capillary or lymphatic vessels) to favor growth and dissemination (1–4). Plasticity of immune cells, referred as the ability of immune cells to be differentially polarized (for example, acquisition of different or opposite phenotypes and functions) within different (micro/macro) environments (1, 2, 4–6) can represent a friend or a foe. Among the most interesting players in the TME regulation of cancer and metastases are myeloid-derived suppressor cells (MDSCs). MDSCs can directly or indirectly (by interacting with several components of both innate and adaptive immunity) contribute to the induction of an immune suppressive environment (7, 8), and angiogenesis (4, 9–11). We will discuss their crosstalk with Natural killer (NK) cells.

NK cells are innate lymphoid cells (ILC) and act as primary effectors of innate immunity, against tumors and virus-infected cells (12). In cancer, NK cells show anergy and hypo-functionality (13–15). NK cells in different tumors have been described by us (4, 16, 17) and other groups (18, 19) to acquire pro-angiogenic phenotype and pro-tumor functions.

MDSCs are recruited and expanded in the TME, in different types of mouse and human cancers (20–24). MDSCs can restrain the CD8^+^ cytotoxic T and NK cells, both of which are anti-cancer, directly influencing the pro-tumor TME. In this review, we will address MDSC-associated angiogenesis and the crosstalk between MDSCs and NK cells, an under-investigated field, and we will focus on relevant cellular and molecular events orchestrating NK-MDSC interactions within the TME, which can impact on tumor insurgence, progression, and angiogenesis.

## NK Cell Phenotype and Functions in Cancer

NK cells are cytolytic and cytokine-producing effector innate lymphoid cells (ILC), representing a first line of defense against virally-infected and transformed cells (12). Spits et al. assigned NK cells as a prototypical ILC family member and classified NK cells as ILC1 subtype, as a consequence of their ability to produce IFNγ, following T-bet and EOMES expression from the ID2^+^ ILC precursor (25). Recently, Vivier et al. put forward that NK cells originate from a separate cell lineage from ILC1. NK cells and ILC1 share the ability to produce IFNγ, following T-bet expression (26). NK cells and ILC1, however, are functionally different: while NK cells are strongly cytotoxic and release perforin, ILC1s cannot release perforin (26).

The field of NK cell biology has expanded well beyond their cytotoxic functions, underlying new roles related to the vast array of cytokines produced by these cells. NK cells are now known to act in immune responses against bacterial (27) and fungal (28, 29) organisms. They have also been shown to play a role in both bone marrow rejection and bone marrow cell engraftment (30). Further NK cell immune regulatory (31) and tissue-regenerative properties (32) have been discovered in viral resistance models.

NK cell cytolytic functions are exerted by perforin and granzyme production and cytokine release. These properties are regulated by a balance between signals from inhibitory receptors (killer Ig-like receptors [KIRs] and the heterodimeric C-type lectin receptor [NKG2A]) as well as activating receptors (the NCRs: NKp46, NKp30, NKp44, and the C-type lectin-like activating immunoreceptor NKG2D), recognizing specific ligands on their cellular targets (12). Peripheral NK cells are predominantly (from 90 to 95%) CD56^dim^CD16^+^ cytotoxic NK cells, that exert their effector functions by perforin/granzyme release and antibody dependent cellular cytotoxicity (ADCC). A minor NK subset, within total circulating NK cells (5–10%), exhibits the CD56^bright^CD16^−^ phenotype and is able to produce high and constant levels of anti-tumor cytokines, such as IFNγ and TNFα (12), CD56^bright^CD16^−^ NK cells are abundant in healthy and neoplastic solid tissues (33).

Pro-angiogenic NK cells have been found in wound healing models (17), a pro-angiogenic NK subset has been also characterized within the developing decidua: decidual (or uterine) NK cells (dNK), that will be discussed later in this review.

Anergic NK cells have been characterized in several tumors, where local immunosuppression resulted in NK cells downregulating NKG2D surface antigen expression, impaired degranulation capabilities, limited abilities to release perforin, granzyme and anti-tumor cytokines (34–38).

We were the first in demonstrating that NK cells in cancer patients (non-small cell lung cancer, NSCLC) (17, 39) colorectal cancer (40) and in malignant pleural effusions (16), show a pro-angiogenic phenotype and function, identified as CD56^bright^CD16^−^VEGF^high^CXCL8^+^IFN^low^ and share several features with the highly pro-angiogenic dNK cells (17, 39, 40). In cancer patients, NK cells mimic behavior of decidual NK, they exhibit a dNK-like phenotype, release pro-angiogenic and pro-metastatic factors and functionally support angiogenesis (4, 16–19, 36, 39, 40). We termed the pro-angiogenic NK cells that are in the TME: “tumor infiltrating NK” (TINKs) and peripheral blood pro-angiogenic NK cells in cancer patients “tumor associated NK” (TANKs) (17).

## Myeloid-derived Suppressor Cell Phenotype and Functions in Cancer

MDSCs identify a heterogeneous immature and mature cell population generated from common hematopoietic progenitor cell. Two major MDSC subsets have been characterized based on their ability a) to phenotypically resemble polymorphonuclear (PMN) cells, termed PMN-MDSCs b) to resemble monocytes, defined M-MDSCs, for their surface markers. Both cell subsets are endowed with potent inhibitory functions against CD8^+^ cytotoxic T cells and NK cells, thus inducing a tolerogenic state and acquiring pro-angiogenic properties (23). In mice, PMN-MDSCs are characterized by CD11b^+^Ly6G^+^Ly6C^lo^ while M-MDSCs by CD11b^+^Ly6G^−^Ly6C^hi^ surface markers. In humans, PMN-MDSCs are identified as CD11b^+^CD14^−^CD15^+^ or CD11b^+^CD14^−^CD66b^+^, and M-MDSCs as CD11b^+^CD14^+^HLA-DR^−/lo^CD15^−^ (20, 41). LOX 1 (Lectin type oxidized receptor-1) represents a more recent marker that has been identified on human PMN-MDSCs, however further confirmation is needed (42).

The immature phenotype of MDSCs is related to the constitutive activation of signal transducer and activator of transcription (STAT)-3, that interferes with the completion of functional cell maturation. The expansion of this subset in tumor patients and tumor-bearing mice is driven also by different factors, such as IRF8, C/EBPβ, Notch, adenosine receptors A2b signaling, and NLRP3 (43). For their immunoregulatory function, the MDSCs requires different pro-inflammatory stimuli, like CSF3, IL-1β, IL-6, and prostaglandin E2 (PGE2), through activation of the NF-κB pathway, as well as of STAT1, STAT6, and cyclooxygenase 2 (COX2) signaling (43), as described more in depth later. Immunosuppressive functions exerted by MDSCs are also mediated through the inducible form of nitric oxide synthase (NOS2) that produces nitric oxide (NO), arginase 1 (ARG1), TGFβ, IL-10, COX2, and indoleamine 2,3-dioxygenase (IDO) (44). PGE2 and HMGB1 are also involved in immune suppression (43). In cancer patients, MDSC expansion in the peripheral blood is correlated with poor clinical outcome and with advanced clinical stage (45–47). Tumors growing in mice lead to the expansion and activation of myeloid cells (48, 49) with similar activities than the human counterparts, resulting in impairment of anti-tumor T cell responses (50).

It has been shown that MDSCs are able to favor the conversion of naive CD4^+^ T cells into Tregs. Retinoids and MDSC-derived TGFβ can promote the trans-differentiation of Th17 cells into Foxp3^+^ Tregs (51).

## MDSC and NK Cell Crosstalk

**Figure 1:**
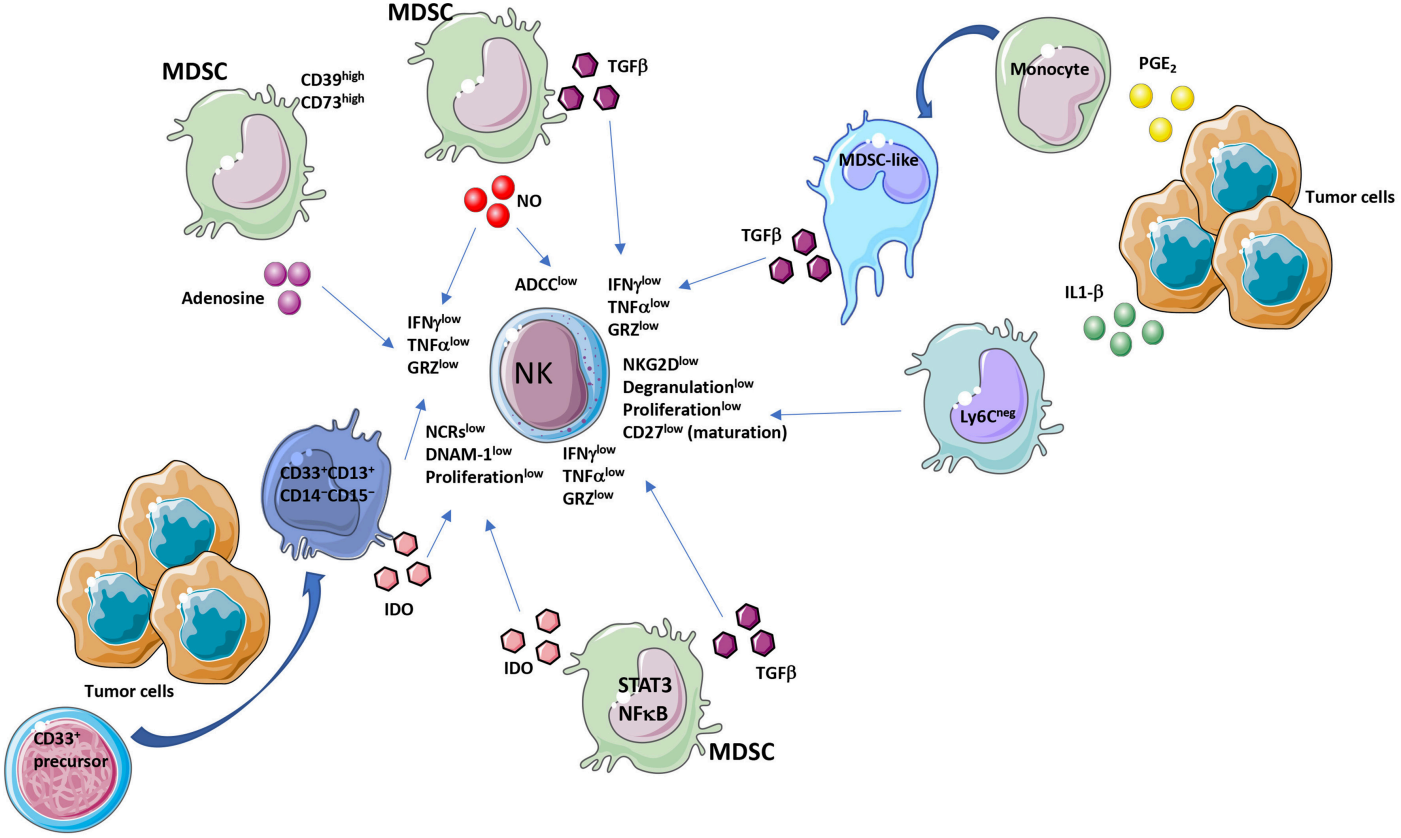
MDSC and NK crosstalk within the tumor microenvironment (TME). Immunosuppressive activities of MDSCs on NK cells act by diverse molecular and cellular mediators. MDSC affect NK cell functionality by several major released factors, among which TGFβ. TGFβ is produced by MDSC or by MDSC-like cells, originated from PGE2 exposed monocytes. Another mediator is IDO produced directly from MDSCs or from a CD33^+^CD13^+^CD14^−^CD15^−^ subset, derived from CD33^+^ precursors. Adenosine from CD39^high^CD73^high^ MDSCs is a further major NK suppressive factor. MDSC effectors decrease NKG2D, NCRs, IFNγ, TNFα, perforin, granzyme levels and ADCC in NK cells.

Immunosuppressive activities by MDSCs have been largely described to be directed toward T cells. Emerging evidence suggests that MDSCs can also interact and regulate the function of other immune cells, including macrophages, DCs and NK cells (7, 8, 52–54). The contribution of MDSCs in regulating NK cell function in tumor-bearing host, still represent a poorly explored topic. MDSCs produce TGFβ which we and others have shown to be a master regulator of NK cell functions in tumors (4, 13, 17, 39, 55–58) (Figure 1). Studies in the literature showed that co-culture of MDSCs with NK cells resulted in impaired tumor cell cytotoxic activity by NK cells and induction of immunotolerance (59, 60). These alterations derived both by MDSC/NK direct interaction (e.g., PDL-1 checkpoint ligand expression and reactive oxygen species production) and via soluble factors (described later in the manuscript). MDSCs have been observed to reduce NK cells tumor suppressive activity (52), and chronic inflammation increases these effects. Several pro-inflammatory cytokines have been reported to orchestrate MDSC/NK crosstalk. Large number of CD11b^+^Gr-1^+^ cells have been found to accumulate in the spleen of tumor-bearing mice and, when adoptively transferred both into tumor-bearing and naïve mice, were able to inhibit NK cell cytotoxicity, by limiting the NK ability to produce perforin *in vivo* and *in vitro* (53). MDSC-mediated NK cell anergy has been associated with the ability of MDSCs to downregulate CD247 expression on the NK cell surface (61). CD247 is a key subunit of natural cytotoxicity receptors (NCRs) NKp46, NKp30, and Fcγ RIII (CD16) (61). MDSCs can inhibit NK cell function by interacting with the NKp30 receptor (62). MDSC/NK cells co-culture results in down-regulation of NKG2D, impaired degranulation capabilities and decreased secretion of IFNγ by NK cells (63). The interaction between MDSCs CD11b^+^Ly6C^med^Ly6G^+^ and NK cells (CD3^−^NK1.1^+^) in the murine pre-metastatic niche has been reported to be critical for metastases establishment (64). The cytotoxicity of NK cells in breast cancer is significantly decreased in the presence of MDSCs, resulting in increased metastatic potential (64). MDSCs inhibit the anti-tumor reactivity of NK cells, promote angiogenesis (65), establish pre-metastatic niches (66), and recruit other immunosuppressive cells (67). MDSC accumulation has been demonstrated to occur, following surgery both in human and mice, which results in dysfunctional NK cells (68–70).

The immune suppressive TME leads to phenotype and functional alterations of several players, including NK cells and MDSCs. Most of soluble molecules within the TME include factors able in shaping NK cell and MDSC response and several of them are shared interactors regulating MDSC/NK crosstalk. Here, we discussed selected soluble factors modulating MDSC/NK cell crosstalk within the TME, as potential candidates to target aberrant phenotype/function endowed with pro-tumor and pro-angiogenic activities.

## Cytokines and Other Mediators in NK and MDSC Regulation

**Figure 2:**
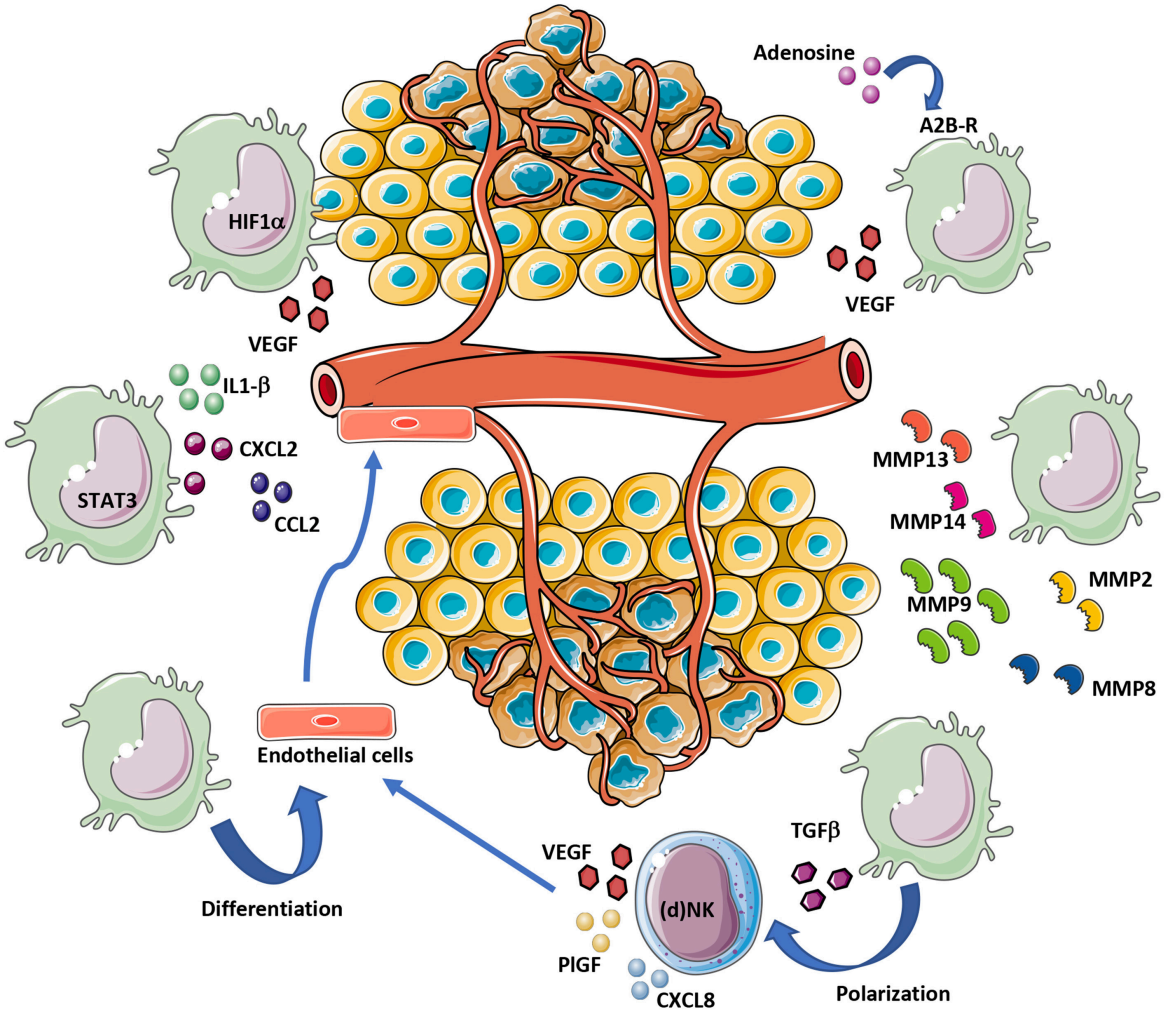
MDSC contribution to tumor angiogenesis. MDSCs can support angiogenesis by different mechanisms. Hypoxia within the TME induce VEGF release directly from MDSCs or indirectly following exposure of MDSCs to TGFβ and adenosine. STAT3 activation in MDSCs also support angiogenesis, via IL1-β, CXCL2, and CCL2 secretion. MDSCs contribute to tumor angiogenesis by ECM remodeling via MMP-2/8/9/13/14 release. Finally, given their cell plasticity, MDSCs can transdifferentiate into endothelial-like cells.

The STAT family are transcription factors that are activated in response to growth factors and cytokines and mediate downstream signaling (71–74). STATs are dysregulated in a broad range of cancer types. STATs have been shown to play diverse roles in innate and adaptive immune cells in the TME (75–77). While STAT2 and STAT4 promote the anti-tumor immune response, STAT3 and STAT6 mediate immunosuppression in the TME, and STAT1 and STAT5 have been implicated in both activation and suppression of the anti-tumor immune response (78). STAT3 activation in an immature MDSC subset, has been found to be crucial for NF-κB activation, resulting in enhanced release of IDO, that limit NK cell proliferation, activation and effector functions (79) (Figure 2). Several studies demonstrated a link between STAT3 blockade, TGFβ inhibition and increased tumor surveillance by NK cells (80, 81). Peripheral and tumor-associated NK cells from STAT3-targeted tumor-bearing mice expressed elevated levels of NK activation markers NKG2D, CD69, Fas ligand (FasL) granzyme B, perforin, and IFNγ, resulting in reduced tumor growth and enhanced survival (80, 81).

IL-2 induced activation of STAT5 leads to NK cell production of perforin, granzyme and IFNγ (82). JAK3-mediated activation of the transcription factor STAT5 is critical in IL-2–stimulated NK cells *in vitro* and Jak3 inhibition has been found in NK cells co-cultured with MDSC isolated from the spleen of tumor-bearing mice associated with reduced STAT5 in NK cells (62). STAT3/STAT5 activation was observed by us in TANKs from colon cancer patients (40). We have shown that treatment with pimozide, a STAT5 inhibitor, reduced endothelial cell capability to form capillary-like networks, inhibiting VEGF and angiogenin production without affecting the levels of TIMP1, TIMP2, and MMP9, indicating that STAT5 is involved in cytokine modulation but not invasion-associated molecules in colon cancer TANKs (40).

MDSCs release TGFβ in the TME (23, 48, 83) (Figure 1). TGFβ exerts anti-tumorigenic effects at early stages, while during tumor progression it acts as crucial orchestrator of angiogenesis, induction of immunosuppression and metastases (84–86). In a murine model of liver cancer, tumor derived MDSCs have been reported to induce NK cell anergy, exhibited as reduced NKG2D expression, degranulation capability, cytotoxicity and IFNγ release *in vitro* and *in vivo*, through membrane-bound TGFβ_1_ (59). Blocking of membrane-bound TGFβ on MDSCs was able to subvert the inhibitory effects on NK cells, demonstrating that MDSC/NK cell-cell contact is necessary to induce MDSC-mediated NK cell anergy.

Elkabets et al. have identified a novel subset of MDSC induced by IL-1β, which lack Ly6C expression (52) (Figure 1). This subset was present at low frequency in tumor-bearing mice in the absence of IL-1β-induced inflammation; while under inflammatory conditions Ly6C^neg^ MDSC were predominant. Ly6C^neg^ MDSC impaired NK cell development and functions *in vitro* and *in vivo* (52) by reduction of NKG2D activating receptor (Figure 1). Another recently identified NK check-point is the IL-1R8 receptor (also known as SIGIRR, or TIR8), which is expressed on human and murine NK cells (87). IL-33, an “alarmin” molecule released upon tissue stress or damage by endothelial and epithelial cells (88, 89), is an IL-1 family member which binds to the ST2 receptor, expressed on immune cells. In murine models, IL-33, depending on the TME, can recruit immune cells with pro-tumor effects, including MDSCs, TAMs, and Tregs, or it can prevent tumor development by stimulating activation and migration of NK and CD8^+^ T cells (88, 89). In humans, IL-33 is associated with poor prognosis in glioma, breast and ovarian cancers, clear-cell renal and hepatocellular carcinoma, while it is correlated with good prognosis in colorectal cancer and lung adenocarcinoma (88, 89).

Nitric Oxide (NO) molecule is a multifunctional gaseous transmitter, playing a key role in inflammation. Paradoxical effects of NO have been documented in cancer, since its anti- or pro-tumor activities are finely tuned by timing, location, and concentration (90, 91). NO production has been largely demonstrated as a key mechanism in MDSC-mediated immunosuppression (90, 92) (Figure 1). Some studies showed that autocrine production of NO by NK cells results in positive effect on NK cell function, and that human NK cells can express endothelial nitric oxide synthase (eNOS) but not inducible nitric oxide synthase (iNOS) (93, 94). In contrast, Stiff et al. recently demonstrated that NO production by MDSCs limits NK cell cytotoxicity by impairing Fc receptor-mediated NK cell function, resulting in altered ADCC (92). They also showed that co-culture of MDSCs with NK cells results in inhibited secretion of IFNγ and TNFα by NK cells (Figure 2).

Prostaglandin E_2_ **(**PGE_2_) is a prostanoid molecule generated by the COX2 inflammatory cascade that have been largely reported to be associated with pro-tumor activities, ranging from induction of tumor cell growth, enhancement of tumor cell migration, invasion, induction of immunosuppression and angiogenesis (95–97). Exposure of monocytes to PGE_2_ results in the generation of a MDSC-like phenotype, together with induction of intracellular signaling pattern, which enables them to suppress NK cell anti-tumor activity in a TGFβ dependent manner (98) (Figure 1). The same effects were observed in NK cells co-culture with freshly isolated CD14^+^HLA-DR^low/−^ M-MDSC from patients with melanoma (98). Selective inhibition of COX limited the accumulation of CD11b^+^Gr1^+^ MDSCs in the spleen, providing improved *in vivo* clearance of NK-cell sensitive YAC-1 cells in murine 4T-1 tumor cells (98). In a mouse model of acute inflammation obtained using zymosan, infiltration of NK cells was an early event with production of IFNγ, which upregulated microsomal PGE synthase-1 (mPGES-1) and COX-1, resulting in sustained PGE_2_ biosynthesis (99). PGE_2_ inhibited lymphocyte function and generated myeloid-derived suppressor cells (99).

Indoleamine 2,3-dioxygenase (IDO) is an intracellular monomeric, heme-containing enzyme able to regulate the tryptophan catabolism into kynurenine (100, 101). Kynurenine production will result in inhibition of proliferation and effector functions in NK and T cells (78, 102–105). MDSCs have been reported as an IDO producer cells within the TME, in both humans and mice. An immature subset of MDSCs, characterized as CD33^+^CD13^+^CD14^−^CD15^−^, has been identified (79, 106). This subset has been found to be induced from CD33^+^ precursor cells that, following co-culture with the human breast cancer cell line MDA-MB-23, result in elevated production of IDO (Figure 1). IDO synthesized by MDSCs blocked NK cell development, proliferation, and activation, resulting in dramatically decreased expression of NCR, NKG2D, and DNAM-1 and by reducing IFNγ release (107, 108) (Figure 1).

As a consequence of hypoxia and inflammation, high levels of adenosine, an immunosuppressive molecule, are released within the TME, (109). Adenosine acts by engaging four subtypes of P1 purinergic or adenosine receptors, A1, A2A, A2B, A3, A2AR, and A2BR, that have been found to be expressed in immune cells (109, 110). A1, A2A, A2B, A3, A2AR, and A2BR mRNA levels dramatically increase in inflammatory cells within the TME (110). Adenosine/adenosine receptor interactions result in subverted immune cell activities, leading to immunosuppression and angiogenesis driven by inflammatory cells (109). The enzyme CD39 converts extracellular ATP to AMP, and CD73 converts AMP to adenosine. MDSCs are able to express high levels of CD39/CD73 in tumor lesions, resulting in higher secretion of adenosine (111, 112) (Figure 2). Adenosine inhibits NK cell anti-tumor activities by blocking granzyme exocytosis, impairing perforin and Fas ligand-mediated cytotoxic activity and limiting IFNγ/TNFα release (113) (Figure 1). CD56^bright^CD16^−^ NK cells produce adenosine through a CD38-mediated pathway, another mechanism to generate extracellular AMP (114). Finally, it has been demonstrated that adenosine signaling is involved in limiting NK cell maturation and that engagement of A2A adenosine receptor (A2AR) acts as a checkpoint in this process (115).

## Decidual NK and MDSCs During Pregnancy: A Possible Onco-Fetal Origin of Pro-Angiogenic Leukocytes

During pregnancy, profound and complex changes occur in the female organism in order to regulate and control the immune response to the fetus, thus conferring tolerance from rejection. This level of regulation in maternal immune system is achieved through coordination and crosstalk of different immune cells, including NK cells, MDSCs, DCs, and Tregs. The dNK cells represent an NK cell subset that has been characterized within the developing decidua and constitutes approximately 70% of the lymphoid cells in the decidua (116, 117). dNK cells have a CD56^superbright^CD16^−^VEGF^high^PlGF^high^ phenotype (58, 116, 117) and are endowed with pro-angiogenic activities, necessary for spiral artery formation. dNK are associated with induction of a tolerogenic environment to host the fetus and permit the correct embryo implantation, both in humans and mice (116, 117). Low levels of dNK cells is associated with miscarriage (17, 116). We have described the expression of angiogenin, in NK from patients with colon cancer (40). Angiogenin was previously reported to be secreted by dNK (118, 119). The TANKs in patients with colon cancer also express MMP2, MMP9, and TIMP, as shared features with dNK cells (116, 120, 121) which could be relevant to the invasive capabilities and proangiogenic functions of colorectal cancer-NK cells (40). Maternal dNK KIR and HLA-C interaction has an effect on birth weight (122), particularly the paternal HLA-C, and correlates with pre-eclampsia and fetal growth restriction (123, 124).

In healthy pregnant women, significant increase in numbers of PMN-MDSCs are detected as compared to non-pregnant controls (125). The raise of PMN-MDSCs mainly occurs in the first trimester (126). Accordingly, reduced PMN-MDSCs are associated with miscarriage (126). The mechanisms involved in this regulation could be related to the release of ARG1, NO, IDO, and indirectly by recruitment and activation of dNK cells and Tregs (127, 128). Serum levels of ARG1, an important effector molecule for PMN-MDSC are significantly reduced in pre-eclampsia patients as compared to healthy pregnant women (129). Behavior of immune cells in tumors might resemble the one in the decidua (4).

## MDSC and Tumor Angiogenesis

**Figure 3:**
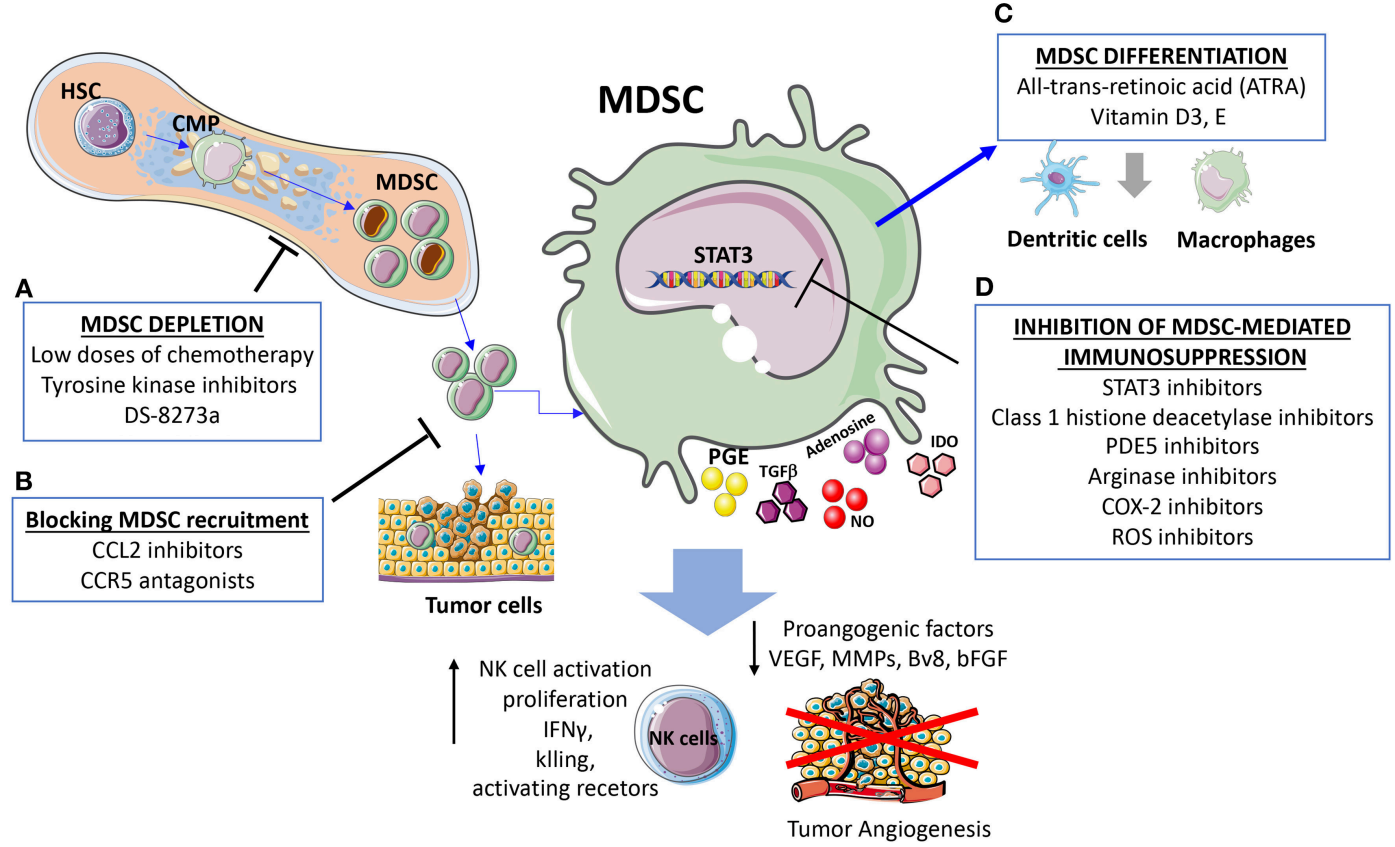
Strategies for targeting MDSC and cross-talk with NK. The presented strategies act simultaneously on MDSC and MDSC-released factors dampening NK cell immunosuppression and induction of angiogenesis. **(A)** MDSC depletion can be induced using low doses of chemotherapy, tyrosine kinase inhibitors, the anti-DR5 monoclonal antibody DS-8273a. **(B)** Strategies blocking MDSC recruitments by CCL2 inhibitors and CCR5 antagonists. **(C)** Differentiation of MDSC into non-immunosuppressive cells induced by all-trans-retinoic acid (ATRA), Vitamin D3, and Vitamin E. **(D)** Inhibition of MDSC immunosuppression can be induced by STAT3 inhibitors, reduction of immunosuppressive agents such as Arginase and ROS, along with attenuation of the inflammatory state.

MDSCs promote tumor progression also through non-immune activities, by stimulating pre-metastatic niche formation, invasion (130, 131) and inducing pro-tumor angiogenesis (132) (Figure 2). In the TME, MDSCs, by production of VEGF, FGF2, Bv8, and matrix metalloprotease (MMP) 9 (MMP9), can trigger and sustain tumor angiogenesis (44, 133) (Figure 3). Co-injection of murine tumors with CD11b^+^Gr1^+^ MDSCs increased intra-tumor vascular density, reduced necrosis, and augmented tumor growth (133, 134). CD11b^+^Gr1^+^ MDSCs cells directly contribute to tumor angiogenesis by producing MMP9 or acquiring endothelial cell properties in TME (133). MDSCs may directly take part in the formation of tumor vasculature by being incorporated into the vessel wall (133, 135) (Figure 3). Several studies have linked MDSC accumulation with an increase in intra-tumor VEGF concentration during disease progression (136). Approaches aiming at reducing levels of circulating MDSCs or in the tumor milieu were associated with decreased angiogenesis and delayed tumor growth (132, 137).

MDSCs can boost angiogenesis and stimulate tumor neovasculature by producing high levels of MMPs (Figure 2), including MMP2, MMP8, MMP9, MMP13, and MMP14 (130, 133, 138). MDSCs from MMP9-knockout mice have a significant reduction in their tumor promoting activity (133). Previous research has indicated that MDSCs with high levels of MMP9, trigger VEGF function by raising its bioavailability (139). In a mouse melanoma model, MDSCs contributed to A2B adenosine receptor-induced VEGF production, increased vessel density and angiogenesis (140, 141).

VEGF in turn stimulates MDSC recruitment, creating a positive feedforward loop. Promoting immunosuppression and angiogenesis (142, 143). MDSCs stimulated by VEGF had stronger immunosuppressive properties than non-stimulated MDSCs (143). VEGF-induced MDSCs stimulate the expansion of other immunosuppressive cells, including FOXP3^+^ Tregs, through a TGFβ-dependent and/or independent pathway (143–145). The relationship between development of resistance to anti-angiogenic therapy with significant MDSC infiltration have been widely demonstrated in several studies (146–148). In agreement with these findings, MDSCs ablation has been reported to have synergistic effects with anti-VEGF/VEGFR treatment in refractory tumors (130, 143). It is now widely accepted that MDSCs interfere with the efficacy of VEGF-targeted therapy, either by secreting large quantities of VEGF that overcome VEGF inhibition, or by activating VEGF-independent pro-angiogenic signaling pathways (149).

The expression of VEGF, MDSCs can modify the TME in a pro-angiogenic manner through the production of several other angiogenic factors and also chemokines which can further enhance MDSCs accumulation within tumors, creating a vicious circle. CCL2, CXCL8, CXCL2, IL-1β, angiopoietin 1 and 2, and GM-CSF have been shown to contribute to MDSC-mediated angiogenesis and require STAT3 for their expression (4, 150–152) (Figure 2). Anti-CCL2 treatment decrease PMN-MDSC and M-MDSC and reduce endothelial cell migration (150, 153, 154). MDSCs promote angiogenesis also via expression of a prokineticin 2, known as Bv8, which plays an important role in myeloid cell-mediated tumor angiogenesis (155). A refractory behavior to anti-VEGF therapy was associated with high number of CD11b^+^Gr1^+^ cells expressing Bv8 in peripheral blood and tumor (156). Thus, it has been suggested that combination of anti-Bv8 antibodies and anti-VEGF may better inhibit angiogenesis and control the tumor growth in anti-VEGF refractory tumors (156, 157). A close expression among molecules associated with angiogenesis: p-STAT3, VEGFA, CK2, and the MDSCs marker CD11b was found in head and neck squamous cell carcinoma (HNSCC) patients (158). Inhibition of JAK2/STAT3 in HNSCC transgenic mouse model reduced MDSC number and suppressed angiogenesis by decreasing VEGFA and hypoxia inducible factor (HIF-1α) both *in vitro* and *vivo* (158) (Figure 2).

Hypoxia, which is a feature of tumor bearing TME, has a crucial role in stimulating HIF-1α mediated signaling. HIF-1 and/or HIF-2 create a proangiogenic TME by inducing the expression of proangiogenic factors (VEGF, ANG-2, PlGF, bFGF, and semaphorin 4D). It was shown that in myeloid cells, HIF-1 activation promotes angiogenesis through VEGF and S100A8 (159) and lead to accumulation of MDSCs positive for the expression of CX3CR1, a CCL26 receptor, in hypoxic tumor regions (111, 160) (Figure 2).

ROS (radical oxygen species) also play an important role in the expansion of MDSCs and augmented levels of these molecules have been shown to stimulate the expression of VEGF receptors on MDSCs and their recruitment in the TME (142, 161).

## Strategies to Target MDSCs and Interfere With NK Crosstalk

The main strategies to target MDSC and consequently their crosstalk with NK cells include: (i) regulation of myelopoiesis and MDSC depletion (tyrosine kinase inhibitors, cytotoxic agents), (ii) enhancement of MDSC differentiation (ATRA, Vitamin A, D3), (iii) inhibition of MDSC recruitment at the site of tumor (CCR5 antagonist, CCL2 inhibitor) (132, 162), (iv) inhibition of MDSC-mediated immunosuppression (STAT3 inhibitors, PDE5, histone deacetylase, NO inhibitors, Arginase inhibitors, ROS inhibitors, COX-2 inhibitors, phenformin, metformin, Polyinosinic-polycytidylic acid) (Figure 3). Here, we will briefly discuss strategies to target MDSC immunosuppression and the effects on angiogenesis and NK cell.

Recent work has provided evidence that relatively low doses of chemotherapy induce MDSC exhaustion (22, 163). Gemcitabine (164), Lurbinectedin (PM01183) (165) 5-azacytidine (166), docetaxel (167), paclitaxel (168), 5-Fluorouracil (169), and doxorubicin (170) exert beneficial effects by reducing MDSC frequencies, increasing responsiveness to immune therapy and enhancing the antitumor activity of activated NK cells (171–174). Similarly, tyrosine kinase inhibitors such as Axitinib, sunitinib, and brutinib, directly target VEGF and/or c-KIT signaling, interfering with tumor-driven expansion MDSC factors such as M-CSF and STAT3 (175–181). In addition to angiogenesis inhibition, sunitinib treatment upregulates NKG2DLs and induces higher cytotoxic sensitivity of tumor cells to NK cells (182–184).

Several studies reported that vitamins D3, A, and E decrease levels of immature MDSC leading to improved anti-tumor activity in the context of immunotherapeutic interventions (185, 186). Vitamin D insufficient and deficient patients had lower NK-mediated cytotoxicity (187), whereas vitamin D receptor (VDR) agonist inhibited selectively ocular hyaloid vasculature angiogenesis in zebrafish models (188). Vitamin E enhance immune responses via reducing ROS levels and inhibition of PGE_2_, COX2, activity mediated through decreasing NO production (189). MDSCs impair NK cell function via production of NO (92), thus, its inhibition offers a strategy for targeting MDSC-NK crosstalk. Promising results on reducing MDSC frequency or increasing their differentiation, were obtained in clinical trials using vitamin A metabolite, *all*-trans-retinoic acid (ATRA), tested alone (190–192) or in combination with IL-2 administration (191) or with a DC vaccine against p53 (193). In preclinical breast cancer models, ATRA improved antiangiogenic therapies by reverting the anti-VEGFR2-induced accumulation of intratumoral MDSCs, decreased hypoxia, and interfered with the disorganization of tumor microvessels (194). Similarly, it was shown that ATRA, suppresses the angiopoietin-Tie2 pathway, inhibits angiogenesis and progression of esophageal squamous xenograft tumors (195). ATRA increased the expression of MICA and MICB in tumor cells, promoting NK cell activation (175, 196), although other studies reported contrasting effects (197, 198).

Blockade of MDSC recruitment at the tumor site inhibits the establishment of an immunosuppressive pre-metastatic niche, via MDSC suppression of NK cells (64). Blocking CCR5/CCR5 ligand interaction by using fusion protein mCCR5–Ig-neutralizing CCR5 ligands, reduced migration, and immunosuppressive potential of MDSCs in the TME and significantly improved survival of tumor-bearing mice (199). In addition, blocking CCL2, which is produced by MDSCs, using specific antibodies can reduce angiogenesis by blocking endothelial cell migration (153).

STAT3 pharmacological inhibition (by peptidomimetics, small molecule inhibitors, platinum agents, curcumin, JAK inhibitors, AG490, Cucurbitacin B) simultaneously blocks angiogenesis and accumulation/suppressive function of MDSC, neutralizing the induction of a tolerogenic/tumor permissive TME, without MDSC depletion (158, 200–202).

JAK/STAT3 inhibitors suppress angiogenesis and reduce MDSCs in the TME through VEGFA and CK2 inhibition (158). Several studies demonstrated a link between STAT3 blockade, TGFβ inhibition and increased tumor surveillance by NK cells (80, 81). Peripheral and tumor-associated NK cells in STAT3-targeted tumor-bearing mice, exhibit higher expression of the NK activation markers NKG2D, CD69, Fas ligand (FasL), granzyme B, perforin, and IFNγ, resulting in reduced tumor growth and enhanced survival (80, 81). Given the STAT3 inhibitors side effects, a STAT3siRNA or decoy STAT3 oligonucleotide inhibitors, such as AZD9150 have been recently developed and combined with immune checkpoint inhibitors, in phase I/II clinical trials (203–205). In similar approach, STAT3 siRNA or decoy oligonucleotides, coupled to CpG oligonucleotides, have been employed to ensure a selective delivery of the drugs to TLR9-expressing myeloid cells (in particular, PMN-MDSC), displaying a decreased immunosuppressive activity (203). Therefore, STAT3 inhibitors provide a potential strategy to reduce immunosuppression activate NK cells and reduce angiogenesis (4).

Class I histone deacetylase inhibitor, entinostat, has been reported to inhibit the immunosuppressive function of MDSC by reducing ARG1, iNOS, and COX2 levels in both M- and PMN-MDSC subsets (206, 207). Vorinostat and entinostat significantly enhanced the expression of multiple NK ligands and death receptors, resulting in enhanced NK cell-mediated cytotoxicity (208).

Several clinical and preclinical mouse model studies, employing PDE-5 inhibitors, such as sildenafil and tadalafil, have demonstrated decreased MDSC accumulation and their immunosuppressive pattern functions by inhibiting iNOS, ARG1, IL4Ra, ROS levels and enabling NK cell anti-tumor cytotoxicity together with activation of anti-tumor response resulting in improved clinical outcome of advanced cancer patients (60, 209–215).

Arginase inhibitors are promising pharmacological agents to treat NK suppression (216) and blocking Arg1 activity in the TME could shift the balance of L-arginine metabolism, favoring T cell and NK cell proliferation (217). In murine studies, injection of the arginase inhibitor hydroxy-nor-arginine (nor-NOHA) or Nω-hydroxy-arginine (NOHA) or genetic disruption of *Arg1* in the myeloid compartment resulted in reduced tumor growth (218–220). In murine syngeneic tumor model, CB-1158, a potent and orally-bioavailable small-molecule inhibitor of arginase, shifted the tumor immune landscape blunting myeloid cell-mediated immune evasion, increasing tumor-infiltrating CD8^+^ T cells and NK cells (182). In colorectal cancer patients undergoing tumor resection, supplementation of arginine prior to surgery resulted in an increase in CD16^+^ and CD56^+^ NK cells infiltrating the tumors (221).

Cyclooxygenase (COX)-2 inhibitors, celecoxib, or nimesulide have been successfully tested in preclinical models for preventing local and systemic expansion of all MDSC subtypes resulting in reduced tumor progression (222–225). On the hand, COX-2 inhibitors induce the expression of NKG2D ligands in cancer cells and increase their susceptibility to NK cell-mediated cell death (226, 227) together with blocking multiple angiogenic and lymphangiogenic such as VEGF-A, VEGF-C/D) (228).

ROS production is one the mechanisms employed by MDSC for immunosuppression (226, 229). In this context, phytochemicals, via their antioxidant property, can activate Nrf2 pathway, that is considered tumor-protective, in particular in the early stages of tumorigenesis. The synthetic triterpenoid C-28 methyl ester of 2-cyano-3,12-dioxooleana-1,9,-dien-28-oic acid (CDDO-Me, also referred to as bardoxolone methyl, RTA402, TP-155, and NSC713200) is a potent Nrf2 activator and has been found to reduce MDSC production of ROS and tumor growth in mouse tumor models (230) and showed a promising anticancer effect in a phase I trial (231). In addition, Nrf2 upregulation, regulates early anti-cancer immune responses and induces the cytokine interleukin-17D (IL-17D), that is overexpressed in highly immunogenic tumor cells and play an important role in immune rejection mediated by NK cells (232, 233). Inducing IL-17D using Nrf2 agonists boost innate immunity and NK recruitment leading to tumor-regression (234, 235). An increasing number of recent reports suggest the abilities of the antidiabetic drugs, phenformin, and metformin to selectively reduce the number -MDSCs and the immunosuppressive functions of MDSC in the TME, through the activation of AMPK (236–240). Phenformin and metformin were able to inhibit immune suppressive activities MDSCs and potentiated the anti-tumor activity of PD-1 blockade immunotherapy (236, 240, 241).

In addition, metformin and phenformin have been widely investigated for their properties in inhibiting angiogenesis and blocking tumor progression (242–244). Several scientific evidences revealed that metformin exerts also strong immunomodulatory effects and contributes to the enhancement of cytotoxic T lymphocyte (245–247) *Polyinosinic-polycytidylic acid [Poly (I: C)* an agonist for pattern-recognition receptors (PRRs), toll-like receptor 3 (TLR3) has been reported to decrease MDSC frequencies in BM, blood, and tumor and abrogate their immunosuppressive, concomitant with an NK cell activation (248–251).

## Conclusions

MDSC are major players in the immunosuppressive scenario in cancer, thanks to their phenotype heterogeneity and critical interaction with several innate immune cells, thus representing a crucial target in oncology. Here we reviewed the interactions of MDSCs with NK cells. The contribution of key cytokines, chemokines and mediators active in this process have been discussed.

We also described the contribution of MDSC on angiogenesis directly or indirectly through interactions with NK and immunosuppressive activities. A parallel of the cancer associated to the decidual counterpart of these cells is discussed, as to propose an onco-fetal origin of the polarization.

In addition to the well-characterized role in immunosuppression, MDSC possess potent pro-angiogenic capabilities, and actively participate in the resistance to VEGF-targeted therapy. Considering the crucial role of MDSC in inducing and regulating a permissive immune TME, in directly contributing to angiogenesis and tumor invasion, several strategies to therapeutically target these cells are currently being tested in clinic. Several pre-clinical studies show that targeting MDSC through multiple approaches helps to increases NK cells tumor activity augment the efficacy of anti-angiogenic therapy.

A better understanding of the link between MDSC-NK immunosuppressive network in TME and their influence on angiogenesis can be translated to new therapeutic targets.

## Author Contributions

DN and AA: design, review and revision of the manuscript, and revision of the figures; LM: writing, review, and revision of the manuscript; DB: writing, review, and preparing figures; AB: design, writing, review, and revision of the manuscript and drafting of the figures.

### Conflict of Interest Statement

The authors declare that the research was conducted in the absence of any commercial or financial relationships that could be construed as a potential conflict of interest.
